# The hydatid cyst fluid protein EgAgB8/1 demonstrates potent immunogenicity by eliciting robust humoral and cellular immune responses in mice

**DOI:** 10.1371/journal.pntd.0014260

**Published:** 2026-05-04

**Authors:** Jie Liu, Jie Xu, Jiahui Xu, Jingqi Zhang, Yinyue Li, Wenjie Huang, Fang Tian, Xinlong He, Ting Zhang, Jun-Hu Chen, Feng Lu

**Affiliations:** 1 School of Basic Medical Sciences, Faculty of Medicine, Yangzhou University, Yangzhou, PR China; 2 University Key Laboratory of Jiangsu Province for Nucleic Acid & Cell Fate Regulation (Yangzhou University), Yangzhou, PR China; 3 Affiliated Hospital of Yangzhou University, Yangzhou University, Yangzhou, PR China; 4 Yangzhou Center for Disease Control and Prevention, Yangzhou, PR China; 5 Yangzhou Central Blood Station, Yangzhou, PR China; 6 National Institute of Parasitic Diseases, Chinese Center for Disease Control and Prevention (Chinese Center for Tropical Diseases Research); National Key Laboratory of Intelligent Tracking and Forecasting for Infectious Diseases; NHC Key Laboratory of Parasite and Vector Biology; WHO Collaborating Centre for Tropical Diseases, Shanghai, PR China; Washington University in St Louis School of Medicine, UNITED STATES OF AMERICA

## Abstract

**Background:**

Cystic echinococcosis (CE), a neglected zoonotic disease caused by the larval stage of *Echinococcus granulosus*, requires effective vaccine strategies for sustainable control. This study evaluated the immunogenic profiles of two antigenic targets: EgAgB8/1, a dominant immunogenic component of hydatid cyst fluid, and Eg-01883, a protoscolex-specific antigen identified through bioinformatic screening.

**Methodology/principal findings:**

*E. granulosus* strains were isolated from infected dogs for genomic DNA extraction. Recombinant proteins rEgAgB8/1 and rEg-01883 were expressed in *E. coli*, purified, and validated by SDS-PAGE and western blot. Initial protein microarray screening identified rEgAgB8/1 as exhibiting markedly higher immunoreactivity with cystic echinococcosis (CE) patient sera compared to the minimally reactive Eg-01883. Based on this finding, subsequent investigation focused on rEgAgB8/1 using a BALB/c mouse immunization model. The recombinant protein elicited potent humoral immunity, with antigen-specific IgG titers reaching 1:16,000, and stimulated significant lymphocyte proliferation. Immunized mouse sera specifically recognized native EgAgB8/1 in hydatid crude antigen preparations, confirming natural antigenicity. Flow cytometric analysis demonstrated that rEgAgB8/1 immunization significantly expanded splenic plasmablasts, memory B cells, and T follicular helper cells. Furthermore, it enhanced IFN-γ production in both CD4⁺ and CD8 ⁺ T cells while maintaining baseline IL-10 ⁺ T cell frequencies, and induced robust T cell memory responses. Statistical analyses were performed using Student’s t-test for comparative evaluation.

**Conclusions:**

These findings establish rEgAgB8/1 as a highly immunogenic antigen capable of eliciting a cellular immune response characterized by dominant IFN-γ production without concomitant IL-10 elevation, alongside durable humoral responses in mice. The comprehensive immunogenicity profile supports further research into its immunological potential against cystic echinococcosis.

## Author summary

rEgAgB8/1 is established as a leading diagnostic antigen and shows promise as a vaccine candidate based on its immunogenic profile, demonstrating a unique capacity to elicit cellular immune responses characterized by robust IFN-γ production without concomitant IL-10 elevation and durable humoral memory-properties essential for effective long-term protection against cystic echinococcosis. In a comparative assessment, Eg-01883 showed markedly lower immunoreactivity with CE patient sera than EgAgB8/1. This contrast suggests differential immunogenicity between these antigens, which may relate to their distinct expression patterns or antigenic properties during infection. This study highlights the superior immunogenicity of rEgAgB8/1 compared to Eg-01883 in our experimental system. While Eg-01883’s low seroreactivity in CE patients suggests limited utility as a broad diagnostic or vaccine antigen in the context tested, its potential value for specific applications (e.g., detection of active protoscolex-driven infections) may warrant further investigation under different conditions. Our findings characterize the immunogenic properties of rEgAgB8/1 and offer a framework for its evaluation as a potential intervention target against this neglected tropical disease, while highlighting the need for protective efficacy studies.

## Introduction

Cystic echinococcosis (CE), a zoonotic infection resulting from the larval form of *Echinococcus granulosus*, affects both humans and animals [1]. The disease is distributed worldwide, with elevated prevalence observed in Eastern Europe, East Africa, the Middle East, and Central Asia-particularly in areas with well-established livestock production systems [2]. Beyond its substantial threat to public health, CE impedes livestock industry development and inflicts considerable economic and healthcare burdens [3]. Vaccination has emerged as a pivotal approach for effective disease control, characterized by its favorable safety profile, absence of chemical residues, and no mandatory withdrawal period in food animals [4]. Current vaccine development efforts against *E. granulosus* encompass conventional, recombinant, nucleic acid, and peptide-based platforms [5]. From early conventional and genetic engineering strategies to recent advances in nucleic acid, peptide, and multivalent vaccines, significant progress has been made in identifying promising protective antigens such as Eg95 [6–8]. However, the intricate host-parasite interactions of this multicellular pathogen have thus far prevented the approval of any vaccine for clinical application.

The EgAgB8 of *E. granulosus* constitutes a highly polymorphic multigene family, with at least five major isoforms identified to date (EgAgB8/1 to EgAgB8/5) [9]. It encodes approximately 8 kDa hydrophobic protein subunits that serve as the core structural components of a high-molecular-weight (∼160 kDa) secretory lipoprotein complex (EgAgB) [10]. This complex is extensively synthesized within the germinal layer and protoscoleces of the hydatid cyst, subsequently released into the hydatid cyst fluid (HCF), and enters the host circulatory system, representing one of the most critical molecules for parasite–host interactions [11]. Its core function is characterized by remarkable immunomodulatory activities: it not only effectively suppresses neutrophil chemotaxis and complement system activation but also impedes dendritic cell maturation and antigen-presenting functions [12,13], while promoting the proliferation and differentiation of regulatory T cells (Tregs) [14]. These mechanisms collectively foster an immune-tolerant microenvironment within the host, ensuring the long-term survival of the parasite. Among this protein family, the EgAgB8/1 isoform has been the most extensively studied due to its highest expression abundance and strongest immunogenicity [15]. As an immunodominant antigen, it is currently the most widely employed recombinant antigen in the serodiagnosis of CE, enabling detection of specific antibodies in patients [16]. Moreover, given its pivotal role at the host--parasite interface, EgAgB8/1 is regarded as a candidate target worthy of exploration for both preventive (intermediate hosts) and therapeutic (definitive hosts) interventions [17]. In-depth functional characterization of this antigen provides a critical theoretical foundation for novel strategies in the control and prevention of echinococcosis. We hypothesized that EgAgB8/1, a dominant immunogenic component of hydatid cyst fluid from *E. granulosus*, exhibits favorable immunogenicity and can effectively elicit specific immune responses in vivo, thereby holding potential as a promising vaccine candidate for the sustainable control of cystic echinococcosis. The protein Eg-01883 from *E. granulosus* corresponds to a specific gene locus designated by this systematic identifier [18]. Bioinformatic analyses predict that it encodes a secreted protease inhibitor, potentially containing domains such as the Kunitz-type serine protease inhibitor motif. It is expressed across multiple developmental stages of the parasite and is likely secreted into the host environment. Owing to its secretory nature and anticipated immunogenicity, Eg-01883 is considered a potential candidate antigen for the serodiagnosis of CE [19]. It may also represent a target for vaccine development strategies. However, these potential applications require further experimental validation to confirm its immunological reactivity and protective efficacy.

Although bioinformatic analyses suggested potential diagnostic and vaccine applications for Eg-01883, its low immunoreactivity with patient sera-revealed in this study by protein microarray-limits its translational utility. In contrast, rEgAgB8/1 showed strong seropositivity, supporting its further evaluation as a vaccine candidate. However, advancing rEgAgB8/1 toward clinical use requires deeper insight into the functional quality and durability of the immunity it induces. Key unanswered questions include whether it promotes short-lived plasmablasts or long-lived plasma cells for sustained antibody production, and whether it can establish a balanced memory T-cell profile-particularly cellular immunity characterized by IFN-γ production-for long-term protection. Moving beyond immunogenicity description, this study functionally characterizes the immune memory landscape driven by rEgAgB8/1. Through comprehensive analysis of antibody-secreting cells, memory B cells, T follicular helper cells, Immunomodulatory cytokines and memory T-cell subsets, we provide critical evidence linking immune induction to potential protection. These findings not only clarify the mechanistic basis of rEgAgB8/1-induced immunity but also support its rational development as a subunit vaccine against CE.

## Materials and methods

### Ethics statement

This study protocol was approved by the Ethics Committee of the National Institute of Parasitic Diseases, Chinese Center for Disease Control and Prevention (NIPD/China CDC, Approval Number: 20180419). All participants were informed that they can leavethe programme at any time, verbal informed consent was obtained from all participants prior to sample collection. For pediatric participants, verbal informed consent was obtained from their parents or legal guardians. All serum samples used in this study were anonymized and did not contain any personally identifiable information; therefore, the use of verbal consent was approved by the ethics committee.

The mice used in this study were obtained from the Experimental Animal Center of Yangzhou University (Yangzhou, China). All animal procedures were performed in accordance with protocols approved by the Laboratory Animal Ethics Committee of Yangzhou University (protocol number 202509030).

### Source of samples and DNA

Samples were collected from two groups: patients infected with *E. granulosus* and healthy individuals. Serum samples from CE patients were obtained from Gansu and Qinghai provinces, while those from healthy controls were collected from Wuxi City, Jiangsu Province. All serum samples were aliquoted and stored at -80°C.

The *E. granulosus* worm samples were isolated from infected dogs and samples were thawed and subjected to mechanical disruption in a sterile mortar. Following the manufacturer’s protocol of a commercial animal tissue DNA extraction kit (GenScript, Nanjing, China), standard procedures including cell lysis, protein digestion, and nucleic acid purification were sequentially performed to obtain high-purity genomic DNA.

### Bioinformatic predictions

The immunogenicity and antigenicity profiles of the target proteins were evaluated through VaxiJen v2.0 (https://www.ddgpharmfac.net/vaxijen/VaxiJen/VaxiJen) and the ANTIGENpro platform (https://scratch.proteomics.ics.uci.edu/). Physicochemical parameters including theoretical isoelectric point (pI), solubility, and instability index were predicted using the ExPASy ProtParam tool. The pI value serves as a guideline for selecting suitable protein purification strategies, whereas the instability index reflects in vitro stability-values below the threshold of 40 indicate a stable protein, with a range of 16.90–38.78 denoting high stability [20]. Furthermore, protein solubility was assessed via the Protein-Sol server (https://protein-sol.manchester.ac.uk/), with a predicted value above the cut-off of 0.45 suggesting favorable solubility characteristics [21].

### Recombinant protein expression and purification

Based on the gene sequences published in the NCBI database, primers were designed using Snap Gene software. Since the *Eg-01883* gene contains introns, a fusion PCR strategy was employed by connecting five gene fragments. Specific primers were designed for each fragment, with the 5’ end of each downstream primer containing a 15-bp sequence complementary to the next upstream primer. The primer sequences were listed in Table 1. *Eg-01883* (EUB63392.1) fragments were amplified from the *E. granulosus* DNA and cloned into the pET28a vector and *EgAgB8/1* (AF143813.1) fragments were cloned into the pET32a vector containing a fused thioredoxin (Trx) tag. The recombinant clones were introduced into *E. coli* Rosetta (DE3) strains for recombinant protein expression and subsequently purified with HIS-select affinity beads. The purified proteins were verified by both SDS-PAGE and western blot, and their concentrations were determined using a BCA Protein Assay Kit (Thermo Fisher, Germany). Endotoxin levels in all purified protein preparations were quantified using the LAL Endotoxin Assay Kit (GenScript, Nanjing, China). The endotoxin concentration was below 0.1 EU/μg for all protein batches used in immunization experiments, ensuring comparable low endotoxin exposure across experimental groups.

**Table 1:** Primer Sequence of *EgAgB8/1* and *Eg-01883.*

Name	Primer Sequence (5, -3)	Length (bp)
EgAgB8/1-F	*gctgatatcggatcc*GATGATGGCCTTACCTCGACG	36
EgAgB8/1-R	*gtggtggtgctcgag*CTATTTACCTTCAGCAACCAACTC	39
Eg-01883–1-F	*atgggtcgcggatcc*GAGATTCCAGTGCGAAGAGGCC	37
Eg-01883–1-R	gatttgatctcctctTAGCCTTGAAATCTGCAAGCTGCCT	41
Eg-01883–2-F	TTATTTATTTTCAGCCCTTCGATGTGTT	28
Eg-01883–2-R	tgccctgaaccaagtCTGCCCGTTTTCATCCTTGGAG	37
Eg-01883–3-F	ACTTGGTTCAGGGCAAAGTTCGATCT	26
Eg-01883–3-R	ggtcaatactccgttCTTGTCCAACTTTGATTTGATCTCT	43
Eg-01883–4-F	AACGGAGTATTGACCGTGGAGG	22
Eg-01883–4-R	cgtattcctctcgAGCGACACTGCGGTCATAGAATCGT	38
Eg-01883–5-F	CTCGAGAGGAATACGTCAGAGAAGAC	26
Eg-01883–5-R	*gtggtggtgctcgag*CGGAATGGGGCCGGTGAT	33

*The homologous arm sequences are indicated in italics, while the complementary 15-bp gene sequences are shown with underlining.

**The restriction enzymes are indicated as italicized and underlined letters.

### Immunization of mice with recombinant proteins

Three groups of 6–8-week-old female BALB/c mice (5 mice per group) were immunized subcutaneously with 50 μg of rEgAgB8/1, Trx, or phosphate-buffered saline (PBS), each emulsified in complete Freund’s adjuvant (CFA; Sigma-Aldrich, St. Louis, MO, USA). CFA was selected for this proof-of-concept study due to its potent immunostimulatory properties, which facilitate robust immune response detection in initial antigen screening. Booster immunizations were administered on days 14 and 28 using the same antigens or PBS emulsified with incomplete Freund’s adjuvant (IFA; Sigma-Aldrich). Blood samples were collected on days 0, 14, 28, and 42; serum was separated and stored at -80°C for subsequent analysis. Mice receiving rEgAgB8/1 served as a protein-induced immune model. For all subsequent immunological analyses (Figs 3-5), samples from each immunization group were pooled from two independent experiments, each consisting of 5 mice per group.

**Fig 1:**
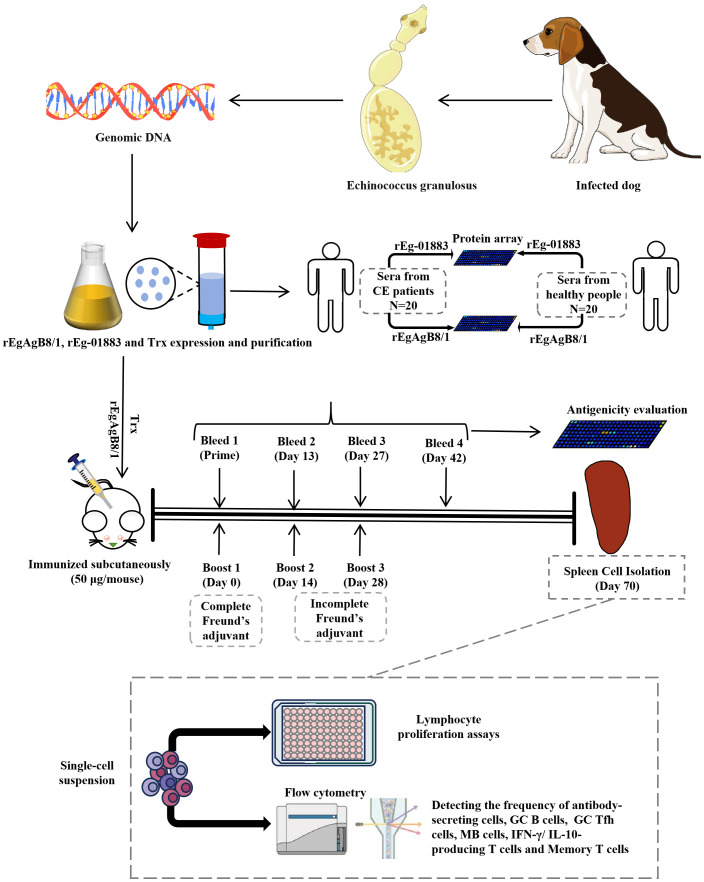
Schematic illustration of the experimental process. Schematic illustration of the experimental workflow. The parasite *Echinococcus granulosus* was isolated from infected dogs, and genomic DNA was subsequently extracted.Recombinant proteins rEgAgB8/1 and rEg-01883 were expressed, purified, and evaluated for immunogenicity through protein microarray analysis using sera from patients with cystic echinococcosis. The antigenicity of rEgAgB8/1 and Trx was further assessed in a mouse immunization model. Flow cytometry was performed to analyze the proportions of antibody-secreting cells (plasmablasts and plasma cells), memory B cells, germinal center (GC) B cells, and T follicular helper (Tfh) cells in the spleens of mice immunized with rEgAgB8/1, Trx, or PBS. Lymphocyte proliferation in response to rEgAgB8/1 and Trx immunization was measured by CCK-8 assay, while serum anti-rEgAgB8/1 antibody levels were analyzed using protein microarrays. Additionally, the frequencies of IFN-γ- and IL-10-producing T cells in mouse.

**Fig 2:**
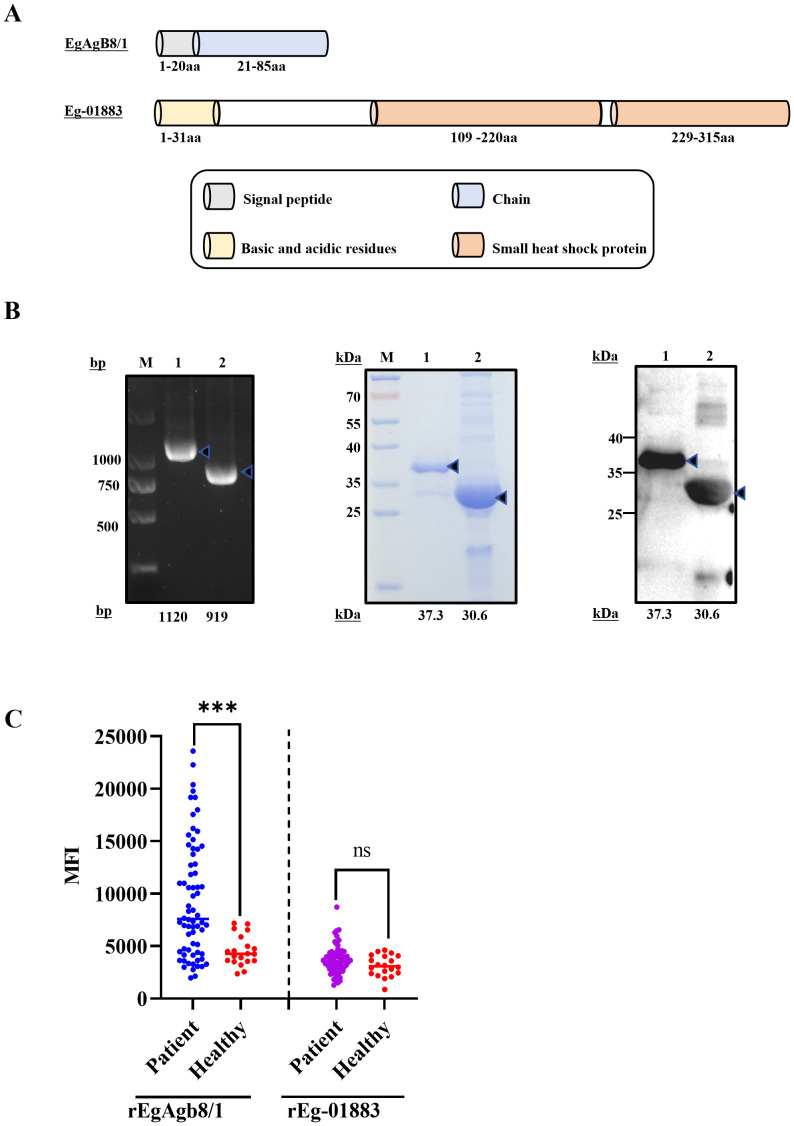
Expression, purification and serological evaluation of rEgAgB8/1 and rEg-01883. **(A)** Schematic diagram of protein structure of *Echinococcus granulosus* protein (EgAgB8/1 and Eg-01883) amino acid (aa) sequences. **(B)** Polymerase chain reaction (PCR, left) amplification for *Eg-01883* (lane 1) and *EgAgB8/1* (lane 2). Coomassie blue-stained SDS-PAGE gel (middle) of eluted, purified and concentrated rEg-01883 (lane 1) and rEgAgB8/1 (lane 2). Western blot analysis (right) of rEg-01883 (lane 1) and rEgAgB8/1 (lane 2) using an anti-HIS rabbit monoclonal antibody. **(C)** Antibody levels of rEgAgB8/1 and rEg-01883 protein in sera from healthy individuals and patients with cystic echinococcosis. The data are shown as the mean ± SD. *P* values were calculated using Student’s t‑test. Significant differences between groups are denoted on the graph: *** *P* < 0.001, nonsignificant (ns), significant differences are indicated (*P* > 0.05).

**Fig 3:**
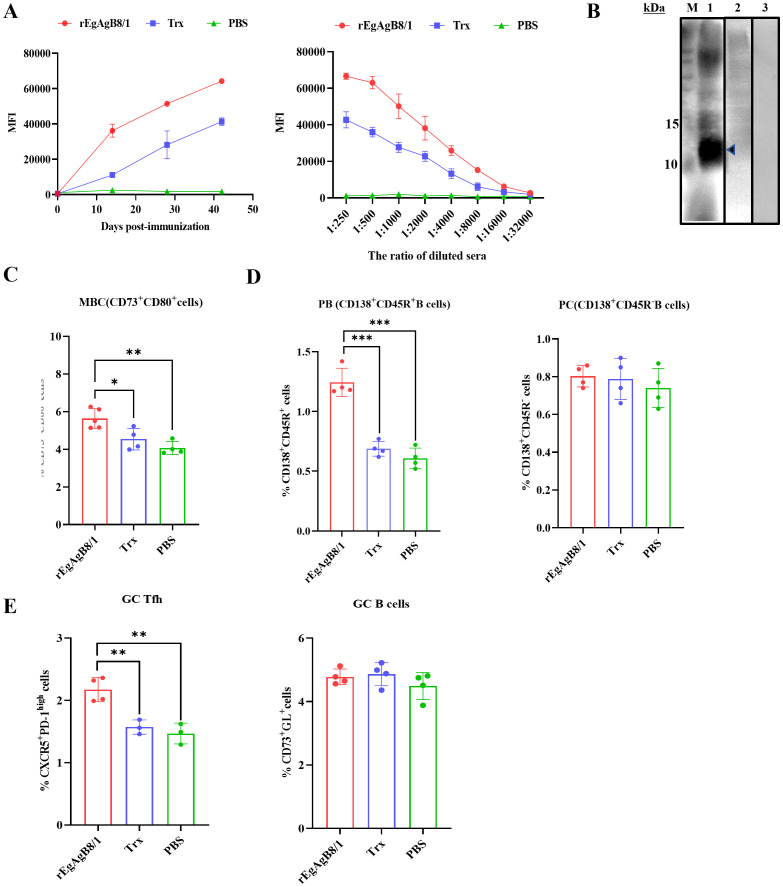
Humoral immune response induced by rEgAgB8/1 and Trx in mice. **(A)** Antibody titers of sera obtained from rEgAgB8/1 and Trx or PBS-immunized mice. Blood samples were collected on days 14, 28, and 42 of post-immunization (left). Sera from immunized mice was arrayed in duplicate in a series of two-fold dilutions (from 1: 250 to 1: 32,000) (right). **(B)** Western blot confirming the presence of rEgAgB8/1 in Echinococcus granulosus lysate using antibodies from the sera of mice immunized with rEgAgB8/1 (lane 1), Trx (lane 2) or PBS (lane 3). **(C)** The percentage comparison of Memory B cells detected by FACS in the spleen of rEgAgB8/1, Trx and PBS-immunized mice. **(D)** The percentage comparison of PB (Plasmablasts) and PC (Plasma cells) detected by FACS in the spleen of rEgAgB8/1, Trx and PBS-immunized mice. **(E)** The percentage comparison of GC Tfh and GC B cells detected by FACS in the spleen of rEgAgB8/1, Trx and PBS-immunized mice. The data are shown as the mean ± SD. Statistical significance was determined by one-way ANOVA with Tukey’s post hoc test for multiple comparisons. Significant differences between groups are denoted on the graph: *P < 0.05, **P < 0.01, ***P < 0.001.

**Fig 4:**
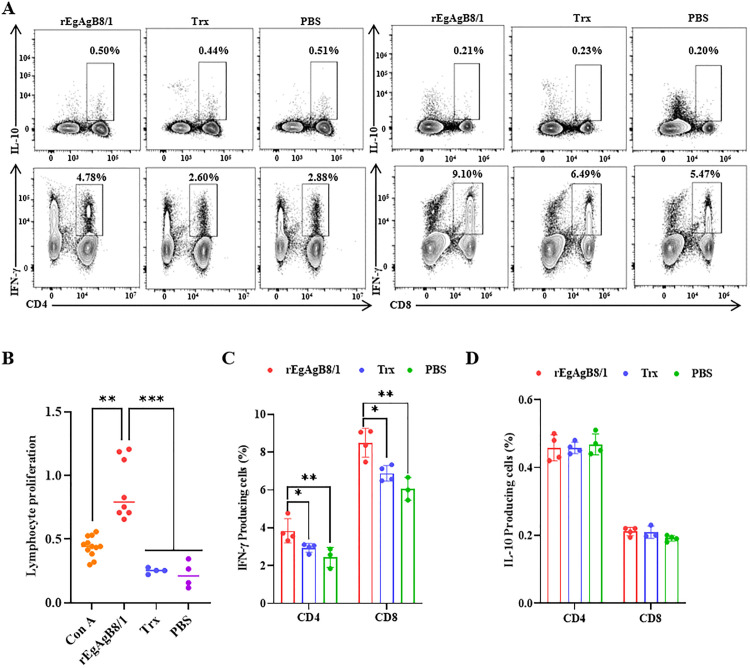
IFN-γ/IL-10-producing T cell responses in rEgAgB8/1-immunized mice. **(A)** Flow cytometry gating strategy for IFN-γ/IL-10-producing T cells upon rEgAgB8/1 antigen. **(B)** Lymphoproliferative activity of splenocytes isolated from experimental groups and PBS group, concanavalin A (Con A) worked as a positive control. Measurement of IFN-γ-producing **(C)** and IL-10-producing **(D)** CD4^+^ and CD8^+^ T cell levels in mice by flow cytometry. Statistical significance was determined by one-way ANOVA with Tukey’s post hoc test for multiple comparisons. Significant differences between groups are denoted on the graph: *P < 0.05, **P < 0.01, ***P < 0.001.

**Fig 5:**
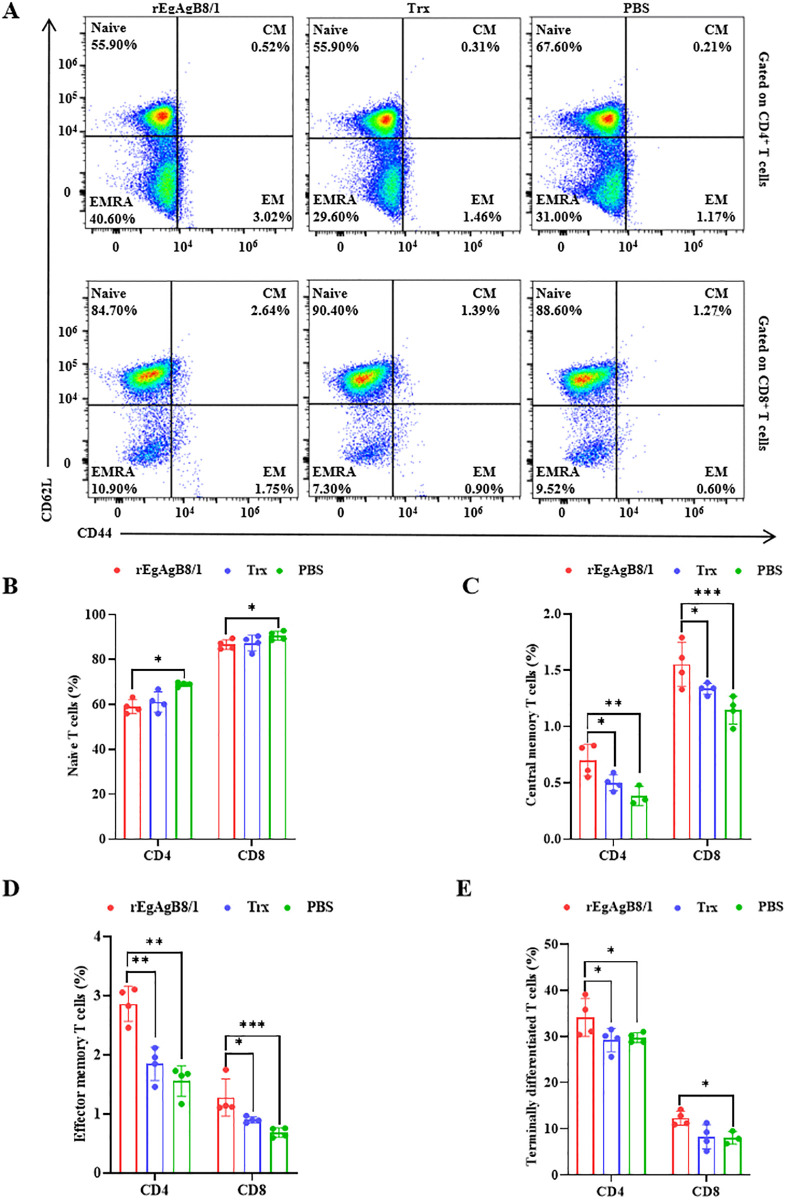
Frequency of memory T cells in response to the stimulation of rEgAgB8/1 antigen. **(A)** The representative gating strategy to identify T naïve cells (T_naïve_, CD44^int/low^ CD62L^+^), T central memory cells (T_CM_, CD44^high^ CD62L^+^), T effector memory cells (T_EM_, CD44^high^ CD62L^−^) and T terminally differentiated cells (T_EMRA_, CD44^int/low^ CD62L^−^). Percentage comparison of T_naïve_ **(B)**, T_CM_ **(C)**, T_EM_ **(D)** and T_EMRA_ **(E)** in CD4^+^ and CD8^+^ T cells after stimulating the splenocytes of rEgAgB8/1, Trx and PBS-immunized mice by rEgAgB8/1 in vitro. Statistical significance was determined by one-way ANOVA with Tukey’s post hoc test for multiple comparisons. Significant differences between groups are denoted on the graph: *P < 0.05, **P < 0.01, ***P < 0.001.

### Protein microarrays

Antigen screening was conducted using nickel-chelate-coated slides for protein microarray analysis [22]. Adhesive microscope slides (Citotest Scientific, Jiangsu, China) were employed to evaluate both the immunoreactivity of proteins expressed in the *E. coli* system and serum antibody titers from immunized mice. Purified EgAgB8/1 and Eg-01883 (1 µL/spot, 20 ng/µL) were spotted in duplicate onto the arrays and incubated at 37°C for 2 hours. Following blocking with 5% BSA in PBST for 1 hour at 37°C, the arrays were incubated with serum under the same conditions. Immunoreactivity assessment utilized sera from CE patients and healthy controls, while antibody titers were determined using serial dilutions (ranging from 1:250–1:32,000) of immune mouse sera. Detection was performed with Alexa Fluor 555-conjugated donkey anti-mouse IgG (1:100, Beyotime) in PBS, and slides were scanned using a LuxScan 10K-B microarray scanner (CapitalBio Technology, Beijing, China).

### Isolation of mouse spleen cells

On day 70 post-primary immunization, mouse spleens were aseptically collected and transferred to a petri dish containing 10 mL of pre-cooled RPMI 1640 (Solarbio, Beijing, China). The tissues were minced with sterile scissors and gently dissociated by pressing through a 70 μm strainer using the flat surface of a syringe plunger until the residual tissue appeared pale. The resulting cell suspension was filtered, washed with RPMI 1640, and centrifuged at 1000 rpm for 5 min at 4 °C. The pellet was treated with 3 mL of cold RBC lysis buffer for 5 min on ice, after which the lysis was terminated by adding complete RPMI medium to a total volume of 10 mL. After two additional washes, cells were counted and adjusted to the target concentration in complete RPMI medium.

### Lymphocyte proliferation assays

Lymphocyte proliferation was assessed via the Cell Counting Kit-8 assay. Splenocytes (5 × 10⁴ cells/well) isolated from mice immunized with rEgAgB8/1, Trx, or PBS were seeded in 96-well flat-bottom plates and stimulated with Con A (2 μg/mL), rEgAgB8/1 (40 μg/mL), or Trx (40 μg/mL). Complete RPMI 1640 medium and Con A served as the blank and positive controls, respectively. After 72 h of incubation at 37 °C with 5% CO₂, cell proliferation was measured using the CCK-8 reagent. The stimulation index (SI) was calculated as follows: SI = [(OD₄₅₀ of stimulated cells − OD₄₅₀ of unstimulated cells)/ OD₄₅₀ of unstimulated cells] × 100%.

### Flow cytometry

Flow cytometric analysis was performed on a Cytoflex S instrument (Beckman Coulter, CA, USA) with antibodies sourced from Biolegend (San Diego, CA, USA) or BD Biosciences (Franklin Lakes, NJ, USA). Splenocytes were depleted of red blood cells and cultured at 5 × 10⁵ cells/mL in RPMI 1640 medium supplemented with 2 mM L-glutamine, 100 U/mL penicillin, 0.1 mg/mL streptomycin, 10% fetal bovine serum (Solarbio), and 50 μM β-mercaptoethanol (Sigma). For surface marker immunostaining, 100 μL of cell suspension was incubated with 0.8 μg/mL of each antibody for 30 min at 4°C, washed, and resuspended in PBS. To evaluate cytokine production, cells were first stimulated for 18 h with 10 μg/mL rEgAgB8/1 or Trx, then treated with PMA/ionomycin in the presence of Brefeldin A for 4 h. Following surface staining for CD3, CD4, and CD8, intracellular IFN-γ and IL-10 were detected using a standard permeabilization procedure. Memory T cell subpopulations were identified based on CD44 and CD62L expression after 24 h of antigen stimulation. Data analysis was conducted using FlowJo v10.

### Statistical analysis

Statistical analyses were performed using GraphPad Prism (version 8.0; GraphPad, USA). Differences between two experimental groups were evaluated using a two-tailed unpaired Student’s t-test, while comparisons among three or more groups were conducted by one-way ANOVA. For all tests, a *P*-value was considered.

The schematic diagram of the whole experimental process is shown in Fig 1.

## Results

### Physicochemical parameters induced by rEgAgB8/1 and rEg-01883

Full-length sequences of rEgAgB8/1 and rEg-01883 were used for physicochemical analysis. First, the instability index of rEgAgB8/1 was found to be below 40, while that of rEg-01883 was slightly above 40, indicating that rEgAgB8/1 is relatively stable, whereas rEg-01883 exhibits certain instability. Second, the solubility values of rEgAgB8/1 and rEg-01883, estimated using the Protein-Sol server (https://protein-sol.manchester.ac.uk/), were 0.82 and 0.35, respectively. This suggests that rEgAgB8/1 is highly soluble (above the threshold value of 0.45), while rEg-01883 is prone to form inclusion bodies. Third, the mean antigenicity scores of rEgAgB8/1 and rEg-01883 were 0.655 and 0.602, respectively, both exceeding the threshold value (0.50), indicating strong immunogenic potential (Table 2). Furthermore, the structural diagrams of both proteins reveal that EgAgB8/1 contains a signal peptide (aa 1–20) followed by a single chain region (aa 21–85) (Fig 2A). In contrast, Eg-01883 features a more complex architecture with a signal peptide (aa 1–31), a central chain domain (aa 109–220), and a C-terminal small heat shock protein domain (aa 229–315), along with distributed basic/acidic residues (Fig 2A). This comparison highlights the structural simplicity of EgAgB8/1 versus the multi-domain organization of Eg-01883, which likely reflects their distinct biological roles.

**Table 2:** Physicochemical parameters and antigenicity prediction of rEgAgB8/1 and rEg-01883 proteins.

Protein name	No. of amino acids	Molecular weight (kDa)	Theoretical pl	Instability index^a^	Solubility	Average antigen index
rEgAgB8/1	85	10.61	9.16	39.97	0.82	0.65
rEg-01883	315	36.07	5.73	43.52	0.35	0.60

*^a^ An instability index threshold of less than 40 is considered stable.

### Expression and purification of the rEgAgB8/1 and rEg-01883

In accordance with bioinformatic predictions, EgAgB8/1 (85 amino acids; theoretical molecular mass: 10.61 kDa) and Eg-01883 (315 amino acids; theoretical molecular mass: 36.07 kDa) were selected for recombinant expression (Table 2). The coding sequences were cloned into pET32a (with Trx tag) and pET28a vectors, expressed in *E. coli* Rosetta (DE3) upon IPTG induction, and verified by SDS-PAGE (Fig 2B). Protein concentrations were determined by the bicinchoninic acid (BCA) assay, yielding approximately 1 mg/mL for rEgAgB8/1 and 2.5 mg/mL for rEg-01883. Western blot analysis using horseradish peroxidase (HRP)-conjugated anti-His antibody confirmed specific recognition of both recombinant proteins (Fig 2B).

### Analysis of the humoral immune response against EgAgB8/1 and Eg-01883 in humans using protein arrays

Serum samples from two distinct groups were analyzed: individuals currently suffering from *E. granulosus*, and healthy individuals with no history of the disease. rEgAgB8/1 demonstrated outstanding diagnostic performance, showing a 63.2.% positive reaction rate with sera from CE patients. Antigen-antibody interactions were further quantified by mean fluorescence intensity (MFI), which revealed significantly stronger immunoreactivity to rEgAgB8/1 in the CE patient group (MFI = 9,320 ± 661) compared to the healthy control group (MFI = 4,551 ± 213). This difference was statistically significant (p < 0.001) (Figs 2C and S1 and S1 Table). In contrast, rEg-01883 showed significantly lower immunoreactivity, with only a 25.0% positive reaction rate in CE patient sera. Furthermore, no statistically significant difference in MFI values was observed between CE patients (3,707 ± 152) and healthy individuals (3,125 ± 147; p = 0.2005) (Figs 2C and S1 and S1 Table).

These findings are consistent with the established biological roles of these antigens. To gain deeper insight into immune response mechanisms, we proceeded to immunize mice with rEgAgB8/1 as the target antigen. This approach enabled us to characterize immune responses, particularly cytokine level changes, and elucidate the specific functions and underlying mechanisms of rEgAgB8/1 in immune protection.

### Humoral immune response induced by rEgAgB8/1 in mice

The antigenicity of rEgAgB8/1 was evaluated by measuring antigen-specific serum IgG titers in mice immunized with rEgAgB8/1, Trx, or PBS using a protein microarray. Preimmune sera were collected to establish baseline levels, and median fluorescence intensity was normalized across arrays. Results showed that anti-rEgAgB8/1 antibody levels in rEgAgB8/1-immunized mice increased progressively from the first immunization, with a more pronounced upward trend compared to Trx- and PBS-control groups. After three immunizations, sera collected on day 42 exhibited dose-dependent reactivity upon serial dilution (1:250–1:32,000), confirming specific antibody titers of up to 1:16,000 (Figs 3A and S2). Importantly, sera from rEgAgB8/1-immunized mice specifically recognized native EgAgB8/1 within hydatid crude antigen preparations, whereas sera from Trx- or PBS-immunized mice showed no detectable binding (Fig 3B). This specific recognition of the native parasite protein further underscores the strong immunogenicity and functional relevance of rEgAgB8/1 as a vaccine candidate.

### rEgAgB8/1 protein induced more antibody-secreting cells and Tfh cells

To assess the B cell immune response elicited by rEgAgB8/1, splenocytes from immunized mice were analyzed by flow cytometry for antibody-secreting cells (ASCs), a term encompassing both short-lived plasmablasts (PBs; defined here as CD138 ⁺ B220 ⁺ cells) and long-lived plasma cells (PCs; defined as CD138 ⁺ B220 ⁻ cells). PBs primarily secrete low-affinity IgM during early immune responses, whereas PCs produce high-affinity antibodies, particularly IgG, for long-term humoral immunity. Additionally, we analyzed T follicular helper (Tfh) cells, specialized CD4 ⁺ T cells that provide critical help to B cells in germinal centers. Immunization with rEgAgB8/1 led to a significant increase in PB frequency compared to Trx- or PBS-treated controls, though no statistically significant difference was observed in the PC subset, indicating a predominant early plasmablast response (Figs 3D and S3). Given the established role of memory B cells (MBCs) in rapidly differentiating into ASCs upon antigen re-encounter—together with the high anti-rEgAgB8/1 antibody levels observed in convalescent CE patients—we further investigated whether antigen recall promotes the differentiation of germinal center B cells toward early MBCs. Splenocytes were stained for the MBC-associated markers CD80, involved in T cell costimulation, and CD73, an ectoenzyme implicated in adenosine production. Phenotypic analysis revealed a distinct CD73 ⁺ CD80 ⁺ MBC population that was significantly expanded in rEgAgB8/1-immunized mice at day 70 post-immunization relative to control groups (Figs 3C and S3). We also evaluated germinal center (GC) B cells and Tfh cells, as GC reactions support affinity maturation and class switching in MBCs. While B220 ⁺ GL7 ⁺ Fas ⁺ GC B cell numbers showed no significant differences across groups, rEgAgB8/1 immunization induced a notable increase in CXCR5 ⁺ PD-1ʰⁱ GC Tfh cells compared to Trx and PBS controls (Figs 3E and S3). Taken together, these findings demonstrate that rEgAgB8/1 vaccination enhances GC Tfh cells, CD73 ⁺ CD80 ⁺ MBCs, and short-lived plasmablasts, highlighting its ability to induce potent antigen-specific B cell and Tfh cell immunity, with a response pattern skewed toward early effector and memory phases.

### IFN-γ-producing T cells contribute to the immune response against rEgAgB8/1

Cellular immunity induced by rEgAgB8/1 was evaluated using splenic lymphocyte proliferation assays. In vitro stimulation with rEgAgB8/1, Trx, or Con A (positive control) revealed that the proliferation rate of splenocytes was significantly enhanced in the rEgAgB8/1 group compared to Trx or PBS controls (Fig 4B). Notably, rEgAgB8/1 triggered a stronger proliferative response than even Con A, whereas Trx alone did not promote detectable proliferation. To further characterize antigen-specific T cell responses, intracellular cytokine staining was performed to quantify IFN-γ- and IL-10-producing cells following rEgAgB8/1 stimulation (Fig 4A). The frequency of IFN-γ T cells was significantly elevated in splenocytes from rEgAgB8/1-immunized mice relative to Trx- and PBS-control groups (Fig 4C). In contrast, no increase in IL-10-producing CD4⁺ or CD8 ⁺ T cells were observed upon rEgAgB8/1 stimulation (Fig 4D). These results indicate that rEgAgB8/1 immunization preferentially activates IFN-γ-producing T cells rather than inducing IL-10-mediated responses, suggesting a cellular immune profile that could contribute to protection against *Echinococcus granulosus* infection, though direct protective efficacy remains to be demonstrated.

### rEgAgB8/1 elicited a specific memory T cell response

Given the crucial role of IFN-γ-producing effector/effector memory cells in sustaining long-term immunity against *E. granulosus* through CD4⁺ or CD8 ⁺ T cell responses, we analyzed the expression of CD62L and CD44 on gated CD4⁺ and CD8 ⁺ splenocytes (Fig 5A). Based on these markers, T cells were classified into four subsets: CD44^int/low^CD62L⁺ naïve (T_naive_), CD44^high^CD62L⁺ central memory (T_CM_), CD44^high^CD62L⁻ effector memory (T_EM_), and CD44^int/low^CD62L⁻ terminally differentiated effector memory (T_EMRA_) cells. In the rEgAgB8/1-immunized group, both CD4⁺ and CD8 ⁺ T cells exhibited significantly elevated proportions of T_EM_ (Fig 5D) and T_CM_ (Fig 5C) subsets, whereas the percentage of T_naive_ cells was markedly reduced compared to the Trx and PBS control groups (Fig 5B). A slight increase in TEMRA cells was also observed in the immunized mice relative to the two control groups (Fig 5E). These phenotypic results confirm that rEgAgB8/1 immunization promotes a broad memory T cell response, characterized by coordinated expansion of T_EM_ and T_CM_ populations across both CD4⁺ and CD8 ⁺ T cell lineages.

## Discussion

Cystic echinococcosis (CE) is a chronic zoonotic helminthiasis that causes substantial economic losses to the livestock industry and poses a public health threat, underscoring the urgency of its prevention and control [23]. However, the complex life cycle and substantial genetic diversity of *E. granulosus* pose considerable obstacles to developing effective interventions [24]. In this context, the identification of highly immunogenic and conserved antigens is essential for informing vaccine development strategies. The initial evaluation of candidate antigens typically focuses on their ability to elicit robust, durable immune responses, which represents a critical first step in assessing their potential for further development. [25,26]. Using immunoinformatic tools such as VaxiJen and ANTIGENpro, we identified EgAgB8/1 and Eg-01883 as antigens with predicted immunogenicity worthy of experimental evaluation [27]. This selection was based on their predicted immunogenicity and reported expression patterns-EgAgB8/1 in HCF and Eg-01883 in protoscoleces-during *E. granulosus* development. Our protein microarray analysis of the humoral immune response to *E. granulosus* infection revealed that EgAgB8/1 exhibited a remarkably high positive reaction rate across all tested sera. In contrast, Eg-01883 showed significantly lower immunoreactivity. The superior performance of EgAgB8/1 may stem from broad exposure: as a major component of HCF, EgAgB8/1 is continuously released into the host circulatory system, ensuring sustained antigenic stimulation [28]. Conversely, the limited immunoreactivity of Eg-01883 with CE patient sera in our assay suggests it may be less effective as a serodiagnostic antigen under the conditions tested. Bioinformatic predictions indicated stage-specific expression for Eg-01883, which could theoretically make it useful for monitoring specific infection phases, but this potential application would require experimental validation beyond the scope of this study. More importantly, in the mouse model, rEgAgB8/1 demonstrated remarkable immunogenicity. It not only induced high titers of specific IgG antibodies but also elicited antibodies that effectively recognized native parasite proteins, reflecting its epitope conservation and structural relevance**.**

Our study found that the proportions of short-lived plasmablasts (PBs) and follicular helper T cells (Tfh) in the spleens of mice in the rEgAgB8/1 immunization group were significantly higher than those in the Trx and PBS control groups. PBs represent an early stage of B cell differentiation responsible for the rapid secretion of low-affinity IgM antibodies, contributing to immediate humoral responses [29]; PCs, through affinity maturation process in the germinal center (GC), produce high-affinity antibodies (such as IgG) that sustain long-term humoral immunity [30]. As major antigenic component of *E. granulosus*, rEgAgB8/1 possesses multiple predicted B cell epitopes, which may efficiently activate naive B cells through the B cell receptor (BCR) and promote their differentiation into PBs and PCs [31]. In addition, the high solubility (Protein-Sol value > 0.45) and stability (instability index 16.9-38.78) of EgAgB8/1 may enhance its antigen presentation efficiency and further promote B cell clonal expansion [32]. Tfh cells secrete cytokines such as IL-21 and provide co-stimulatory signals including CD40L, directly assisting B cells in undergoing somatic hypermutation and class switch recombination in the GC [33]. The increase in the proportion of Tfh cells indicates that rEgAgB8/1 may activate the differentiation of CD4^+^ T cells into Tfh cells through the presentation of antigen peptide-MHC II complexes by dendritic cells (DCs) [34]. This Tfh-B cell interaction is central to GC formation and the production of high-affinity antibodies [35]. Similarly, our results demonstrated a significantly higher proportion of memory B cells (MBCs) in the spleens of mice immunized with rEgAgB8/1 compared to the Trx and PBS control groups. This indicates that rEgAgB8/1 immunization effectively primes the immune system and establishes a pool of MBCs under steady-state conditions [36]. Upon antigen re-encounter, these pre-existing MBCs are rapidly activated and differentiate into antibody-secreting plasma cells, leading to the swift production of high-titer antibodies [37]. This finding confirms the ability of rEgAgB8/1 to induce durable B cell memory, a feature of interest for antigens considered in vaccine development. The pathological feature of hydatid disease is chronic cyst formation, and its immune evasion mechanisms include the inhibition of host response favoring IFN-γ production and the induction of regulatory T cells (Treg) [38]. The immune profile induced by rEgAgB8/1 offers insights into its potential relevance for hydatid disease intervention While the PC population showed no significant expansion, the robust plasmablast response and elevated antigen-specific IgG titers suggest active humoral immunity. The induced antibodies are capable of neutralizing parasite antigens in cyst fluid, counteracting their immunosuppressive effects [39], and mediating parasite clearance through antibody-dependent cellular cytotoxicity (ADCC) or complement activation pathways [40]. The GC reaction driven by Tfh cells is the basis for the production of long-lasting protective antibodies [41]. In natural infections, *E. granulosus* may inhibit GC formation by secreting immunomodulatory molecules (such as antigen B), potentially limiting antibody affinity maturation [13]. Our findings suggest that immunization with rEgAgB8/1 may circumvent this immunosuppression by enhancing Tfh responses, promoting GC reactions and supporting the generation of high-affinity antibodies. The activation of MBCs indicates that rEgAgB8/1 immunization can induce immunological memory, which is crucial for preventing reinfection of hydatid disease [42]. In endemic areas, the host may be repeatedly exposed to the parasite, and the rapid response ability of MBCs can shorten the immune response window period and reduce the risk of cyst formation.

Serological reactions indicate that rEgAgB8/1 may be the target of immunologically active antibodies. Moreover, sera from immunized mice maintained high antibody titers two months post-vaccination. When formulated with a suitable adjuvant, rEgAgB8/1 effectively induced elevated antibody levels, though the durability and profile of the cellular immune response warrant further investigation. Freund’s adjuvant was used in this study to demonstrate the robust immunogenicity of rEgAgB8/1. While unsuitable for clinical translation, it established that the antigen can elicit high-titer antibodies, IFN-γ-driven T cell responses, and immune memory. In contrast, clinically relevant adjuvants may shape the immune profile differently: alum tends to promote Th2-biased humoral immunity, whereas MPLA could better sustain the Th1 response observed here, and saponin-based adjuvants might further enhance cellular immunity. Future work should aim to identify a clinically acceptable adjuvant formulation that preserves these favourable immune features while meeting safety requirements for vaccine development. An effective vaccine should concurrently activate humoral immunity-driving B cells to produce specific antibodies-and cellular immunity, enabling T cells to generate antigen-specific lymphocytes for comprehensive protection against CE [43]. Sufficient amounts of *Echinococcus*-specific antibodies, as well as CD4^+^ and CD8^+^ T cells, can mediate protective immunity during the blood stage [44]. Our results show that immunization with rEgAgB8/1 is capable of inducing IFN-γ-producing T cells, rather than IL-10-producing T cells, to participate in the cellular immunity mediated by rEgAgB8/1 against hydatid infection [45]. Several studies have repeatedly demonstrated that IFN-γ is crucial for parasite clearance and protective efficacy [46].

While IFN-γ can be produced by multiple immune cell types, memory T cells are recognized as central mediators of sustained IFN-γ responses during recall immunity [47]. In this study, we observed that both CD4⁺ and CD8 ⁺ T cells contributed significantly to IFN-γ production following rEgAgB8/1 immunization. Phenotypic analysis revealed a distinct memory T-cell profile in immunized mice, with both CD4⁺ and CD8 ⁺ T-cell compartments exhibiting significant expansion of effector memory (T_EM_, CD44ʰⁱCD62L⁻) and central memory (T_CM_, CD44ʰⁱCD62L⁺) subsets compared to controls. These findings differ from an earlier report suggesting that CD4 ⁺ T cells serve as the primary IFN-γ producers in response to rEgAgB8/1. The broad memory T-cell expansion observed here may reflect the dynamic nature of the immune response during later stages of immunization. While CD4 ⁺ T cells may dominate early IFN-γ production, the coordinated expansion of both CD4⁺ and CD8 ⁺ memory subsets at later time points likely contributes to a more sustained and robust IFN-γ response, possibly through compensatory mechanisms involving T-cell cross-talk or cytokine-mediated amplification [48]. The concurrent increase in both T_EM_ and T_CM_ subsets across CD4⁺ and CD8 ⁺ T cells suggests that rEgAgB8/1 immunization promotes a comprehensive memory T-cell pool, capable of mounting both immediate effector functions and long-term immunity. This balanced memory profile may underlie the enhanced protective immunity observed in immunized hosts.

While this study supports the immunogenic profile of rEgAgB8/1, several limitations must be acknowledged. The most significant limitation of this study is the absence of parasite challenge experiments to assess protective efficacy. While we have comprehensively characterized the immunogenic profile of rEgAgB8/1, demonstrating its ability to induce robust humoral and cellular immune responses, we cannot conclude from these data alone that these responses will translate into reduced cyst burden, impaired protoscolex viability, or protection against infection. The correlation between immunogenicity and protection, while encouraging, requires direct validation in appropriate challenge models. Furthermore, immune analyses were confined to splenic responses without assessing local immunity at cyst sites, and cytokine profiling was limited to IFN-γ and IL-10, which does not allow full characterization of T helper polarization. The use of a Trx fusion tag and Freund’s adjuvant, though methodologically justified here, may influence immune readouts and limits direct clinical extrapolation. Finally, evaluation was restricted to a single EgAgB8 subtype, leaving questions regarding cross-strain efficacy and long-term safety unresolved. These limitations underscore the need for future studies employing challenge models, clinical-grade formulations, and broader antigenic and safety assessments.

## Supporting information

S1 Fig Antibody levels of rEgAgB8/1 and rEg-01883 protein in sera from healthy individuals and patients with cystic echinococcosis. (TIF)

S2 Fig Humoral immune response induced by rEgAgB8/1, Trx and PBS in mice. (TIF)

S3 Fig Flow Cytometry Gating Strategy. (TIF)

S1 Table Prevalence of IgG antibodies against the rEgAgB8/1 and rEg-01883 proteins. (PDF)

S2 Table The fluorophore and clone number for all flow cytometry detection antibodies used in this study. (PDF)
