# Supplementary figures and images for: The hydatid cyst fluid protein EgAgB8/1 demonstrates potent immunogenicity by eliciting robust humoral and cellular immune responses in mice
Source: PLoS Negl Trop Dis. 2026 May 4;20(5):e0014260. doi: 10.1371/journal.pntd.0014260 (PMC13138655; doi:10.1371/journal.pntd.0014260)

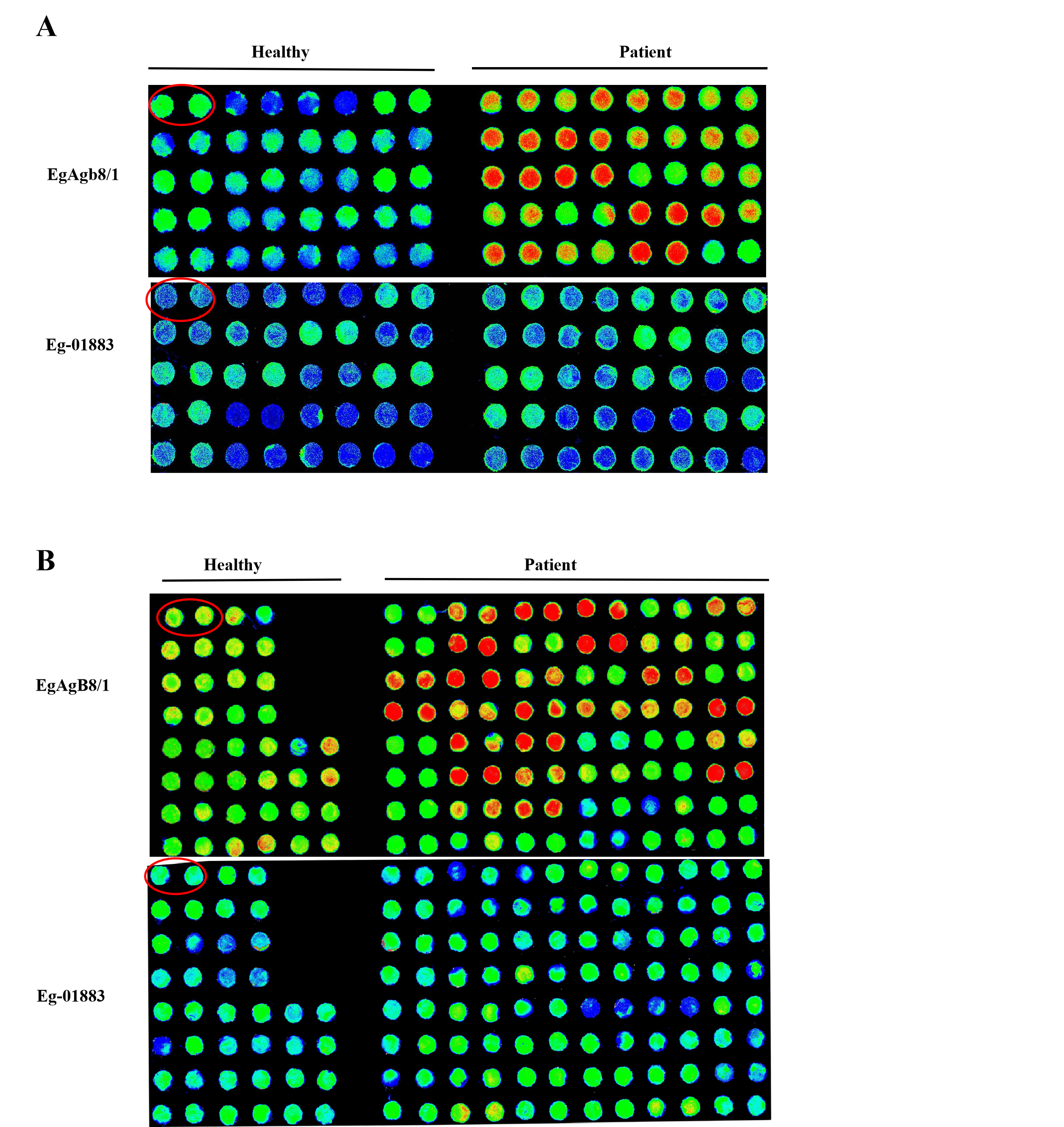

Supplement: S1 Fig — (TIF) [file pntd.0014260.s001.tif]

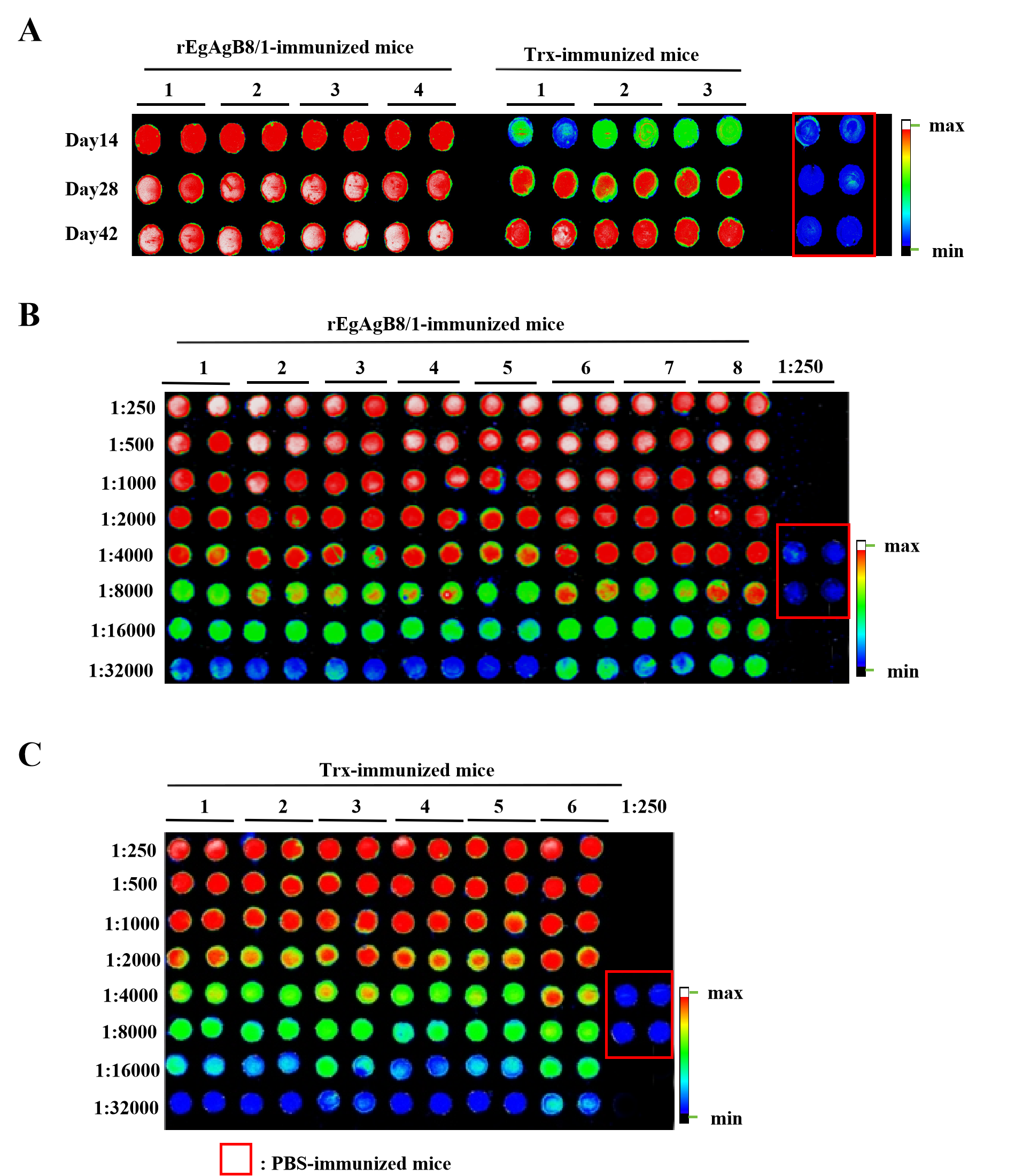

Supplement: S2 Fig — (TIF) [file pntd.0014260.s002.tif]

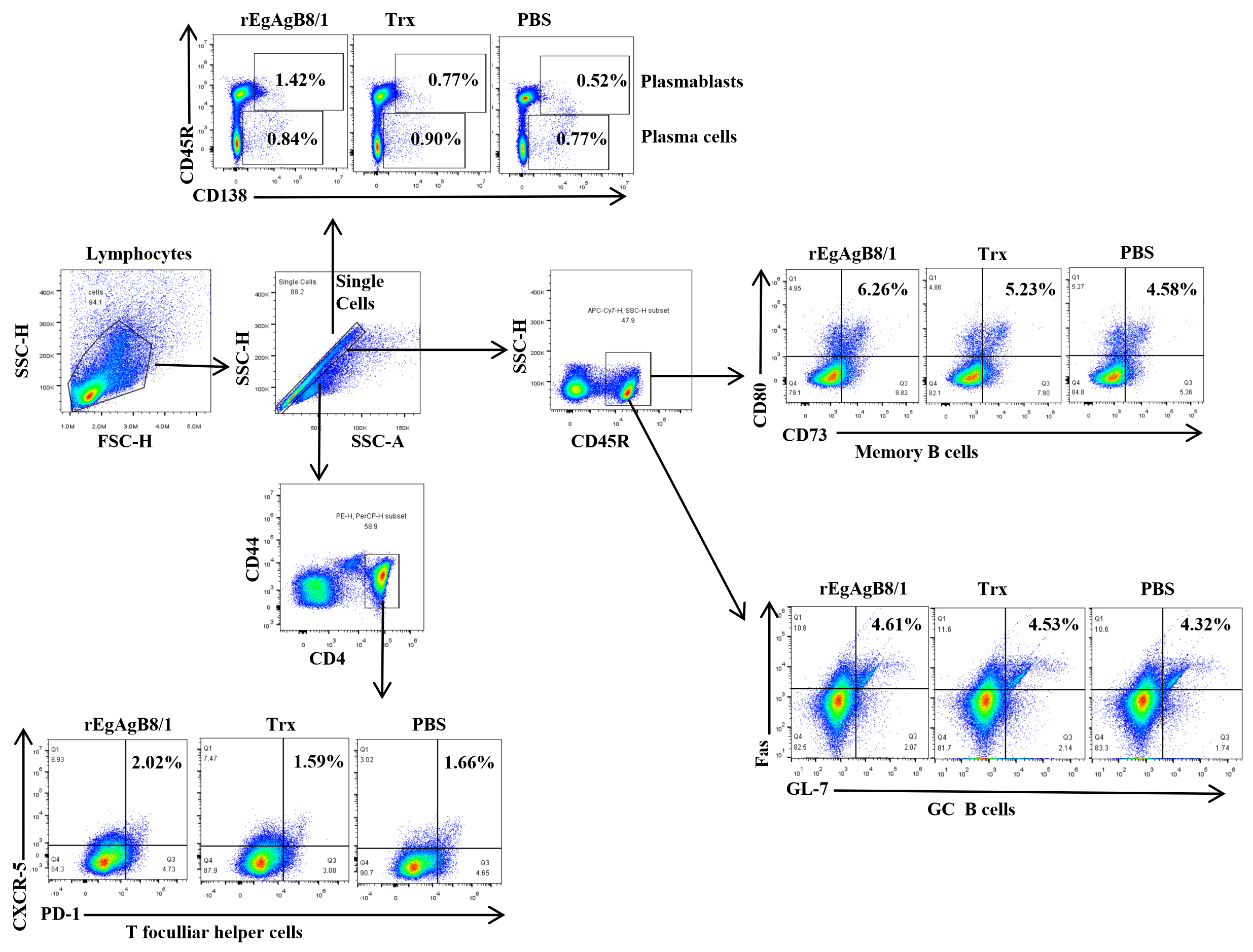

Supplement: S3 Fig — (TIF) [file pntd.0014260.s003.tif]
