# Supplementary material for: The hydatid cyst fluid protein EgAgB8/1 demonstrates potent immunogenicity by eliciting robust humoral and cellular immune responses in mice
Source: PLoS Negl Trop Dis. 2026 May 4;20(5):e0014260. doi: 10.1371/journal.pntd.0014260 (PMC13138655; doi:10.1371/journal.pntd.0014260)
Supplement: S1 Table — (PDF) [file pntd.0014260.s004.pdf]

Table S1. Prevalence of IgG antibodies against the rEgAgB8/1 and rEg-01883 proteins.

Antigen	Sample	No. positive	No. negative	Sensitivity a (%)	Specificity b (%)	Mean fluorescence intensity (MFI)		*P* value
						MFI±SEM	Highest MFI<br>Lowest MFI	
rEgAgB8/1	CEI	43	25	63.2		9320±661	23590<br>1967	< 0.001
	HI	1	19		95.0	4551±213	7171<br>2365	
rEg-01883	CEI	17	51	25.0		3707±152	8718<br>1277	0.2005
	HI	1	19		95.0	3125±147	4610<br>868	

*a Sensitivity=Positive serum number/ Patient serum number × 100%
**b Specificity=Negative serum number/ healthy individual serum number × 100%
