# Supplementary material for: The hydatid cyst fluid protein EgAgB8/1 demonstrates potent immunogenicity by eliciting robust humoral and cellular immune responses in mice
Source: PLoS Negl Trop Dis. 2026 May 4;20(5):e0014260. doi: 10.1371/journal.pntd.0014260 (PMC13138655; doi:10.1371/journal.pntd.0014260)
Supplement: S2 Table — (PDF) [file pntd.0014260.s005.pdf]

| Antibody/dye | Fluorochrome | Catalog # | Company |
|---|---|---|---|
| T- and B-Cell Activation Antigen (GL7) | FITC | 562080 | BD Pharmingen |
| CD45R(RA3-6B2) | APC-CY7 | 552094 | BD Pharmingen |
| CD138(281-2) | APC | 558626 | BD Pharmingen |
| CD93 (Early B Lineage) (AA4.1) | PE | 558039 | BD Pharmingen |
| CD3 | FITC | 100204 | BioLegend |
| CD4 | PE | 100408 | BioLegend |
| CD44 | Percp | 103035 | BioLegend |
| CD185 (CXCR5) | APC | 145505 | BioLegend |
| CD279 (PD-1) | PE-CY7 | 135215 | BioLegend |
| CD95 (Fas) | PE | 152607 | BioLegend |
| CD80 | PE | 104707 | BioLegend |
| CD73 | FITC | 127219 | BioLegend |
| IFN-γ | PE | 560660 | BioLegend |
| IL-10 | PE | 561059 | BioLegend |
